# Causal relationship of interferon-γ and interleukin-18 upstream of intervertebral disc degeneration pathogenesis: a two-sample Mendelian randomization study

**DOI:** 10.3389/fneur.2024.1420942

**Published:** 2024-06-19

**Authors:** Fang Gao, Chen Deng, Zhiqiang Wang, Beiyang Wang, Junqiao Lv, Lin Sun

**Affiliations:** Third Hospital of Shanxi Medical University, Shanxi Bethune Hospital, Shanxi Academy of Medical Sciences, Tongji Shanxi Hospital, Taiyuan, China

**Keywords:** IVDD, serum metabolites, Mendelian randomization, inflammatory cytokine, IFN-γ, IL-18

## Abstract

**Introduction:**

Intervertebral disc degeneration (IVDD) is a complex disease caused by genetic and environmental factors, but its pathogenesis is still unclear. Although studies of inflammatory cytokines have been used in recent years to unravel the biological mechanisms of a variety of diseases, such analyses have not yet been applied to IVDD. Therefore, we used a Mendelian Randomization approach to explore the potential mechanisms underlying the pathogenesis of IVDD.

**Methods:**

We obtained GWAS data from publicly available databases for inflammatory cytokines and IVDD, respectively, and explored the causal relationship between individual inflammatory cytokines and IVDD using instrumental variable (IV) analysis. We primarily used IVW methods to assess causality, while sensitivity, heterogeneity and multidirectionality analyses were performed for positive results (*p* < 0.05). All analyses were performed using R software.

**Results:**

In our study, we performed a two-sample MR analysis of 41 inflammatory cytokines to identify metabolites causally associated with IVDD. Ultimately, 2 serum metabolites associated with IVDD were identified (pval<0.05), IFN-γ and IL-18. sensitivity, heterogeneity, and Pleiotropy test analyses were performed for all results.

**Conclusion:**

Our study identified a causal relationship between IFN-γ and IL-18 and IVDD. It is valuable for the monitoring and prevention of IVDD and the exploration of targeted drugs. However, more evidence is needed to validate our study.

## Introduction

Intervertebral disc degeneration (IVDD) is a major pathological process implicated in low back pain (LBP) and is a prerequisite to disc herniation (1, 2). It is a major contributing factor for discogenic LBP, causing significant global disability (3–5). The biological mechanisms of IVDD are still unknown and complex to researchers currently (6). Current treatment strategies for IVDD are not satisfactory due to the lack of understanding of the mechanisms of IVDD.

Inflammation is a complex biological response to harmful stimuli such as pathogens, damaged cells, or irritants (7). During the inflammatory response, various types of inflammatory cells are coordinated to release inflammatory cytokines as soluble mediators that are involved in numerous biological behaviors (8, 9). We note that IVDD is a complex biological response that involves a large number of inflammatory cytokines. Inflammatory cytokines such as vascular endothelial growth factor (VEGF), tumour necrosis factor α (TNF-α), interleukin 1 (IL-1), and chemokine (C -C motif) ligand 2 (CCL2/MCP-1) are involved in IVDD (10–13). However, studies on the relationship between inflammatory cytokines and IVDD are still scarce.

Mendelian randomization (MR) is a powerful tool for making causal inferences in epidemiological studies (14, 15). MR refers to an analytic approach that uses genetic variants as instrumental variables (IVs) to assess the causality of an observed association between a modifiable exposure or risk factor and a clinically relevant outcome (16, 17). This approach has been used in the study of various diseases and has led to significant advances in our understanding of the causal relationship between risk factors and disease outcomes (17–19).

The aim of this study is to explore, through MR, the relevant inflammatory cytokines in a causal relationship with IVDD, and discussing the possible biological mechanisms based on the available evidence.

## Materials and methods

**Figure 1:**
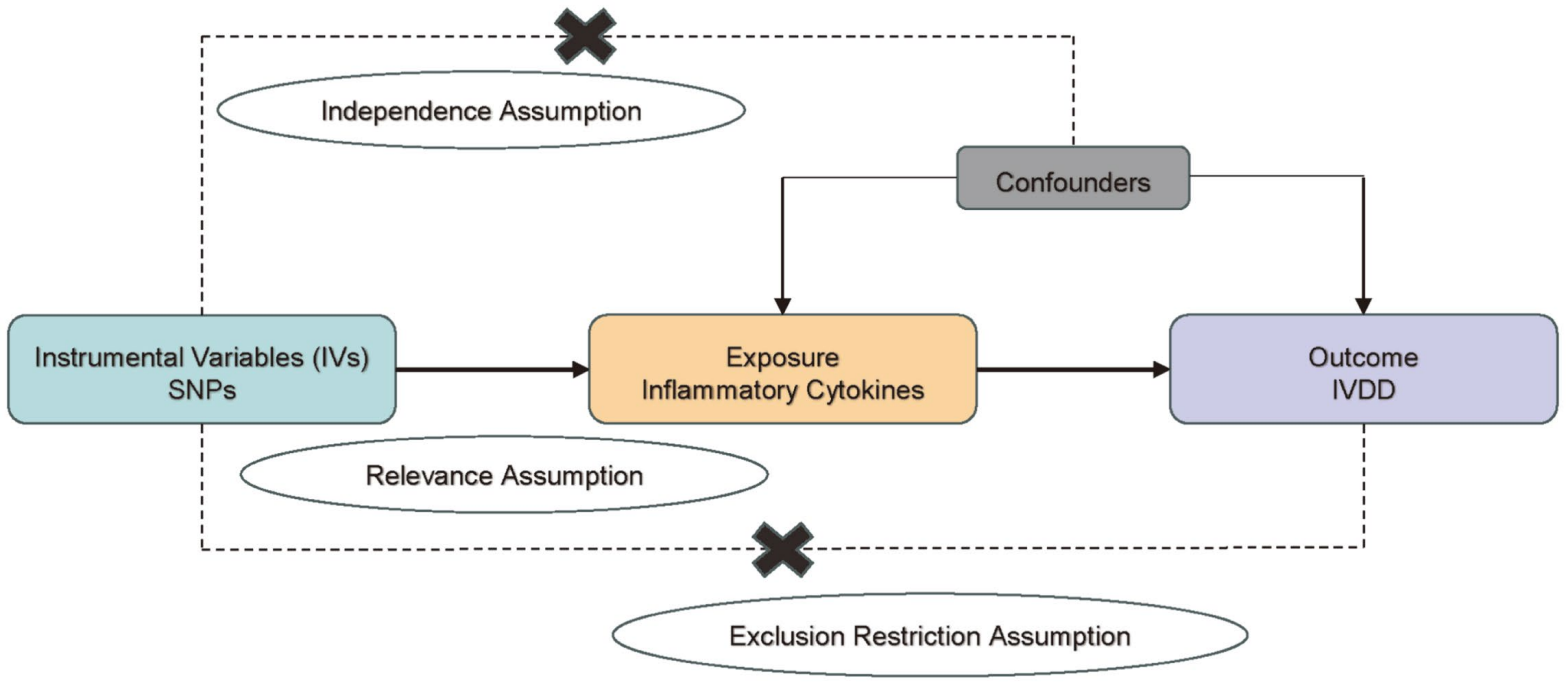
Design of the study based on three basic assumptions.

Mendelian randomization is a statistical method that uses genetic variants as instrumental variables to estimate the causal effect of an exposure on an outcome (15). The method is based on three core assumptions: 1. Relevance Assumption: The genetic variants (instrumental variables) used in MR analysis must be associated with the exposure of interest (e.g., cytokine levels). This means that the chosen SNPs must have a statistically significant effect on the levels of the cytokines we are studying. To address this, we selected SNPs with robust associations with the cytokines from genome-wide association studies (GWAS) and ensured their relevance through statistical validation (20). 2. Independence Assumption: The genetic variants used as instruments should not be associated with any confounders that influence both the exposure and the outcome. This assumption ensures that the observed association between the genetic variants and the outcome is not due to confounding factors. We conducted sensitivity analyses, such as the use of negative control outcomes and additional GWAS data, to check for potential pleiotropy and confounding, thereby supporting the independence assumption (21, 22). 3. Exclusion Restriction Assumption: The genetic variants affect the outcome only through the exposure and not through any other pathway. This means that the SNPs should influence the risk of IVDD solely through their effect on cytokine levels, without any direct effect on the outcome. To test this, we used methods such as MR-Egger regression and the weighted median approach to detect and correct for potential violations of this assumption, ensuring that our results are not biased by direct effects (14, 15, 23). Based on these three assumptions, we have designed the study (Figure 1).

### Data sources

The source of inflammatory cytokines is derived from the GWAS report by Kalaoja et al. (24). Which reports the most comprehensive exploration to date. The results reported here are the meta-analysis summary statistics for the 41 inflammatory cytokines, which included 8,293 subjects, without fitting BMI as a covariate in the model (as was done in the previously published GWAS). Results are filtered such that only SNPs with results for 2 or more of the 3 cohorts remain.^1^ IVDD data from FINNGEN where ncase = 37,636, ncontrol = 270,964, Number of SNPs = 20,175,454. The data are available at https://www.finngen.fi/fi. We defined this phenotype based on the International Classification of Diseases, 10th Revision (ICD-10), which is widely used in previous studies.

No ethical approval was necessary for the publicly available de-identified data.

### Instrumental variables

The following selection criteria were used to select the IVs: (1) single nucleotide polymorphisms (SNPs) associated with each Serum metabolites at the locus-wide significance threshold (*p* < 1.0 × 10^−6^) were selected as potential IVs; (2) 1000 Genomes Project European sample data were used as the reference panel to calculate the linkage disequilibrium (LD) between the SNPs, and among those SNPs that had R^2^ < 0.001 (clumping window size = 10,000 kb), only the SNPs with the lowest *p*-values were retained; and (3) SNPs with minor allele frequency (MAF) ≤ 0.01 were removed. Regarding the nature of the SNPs analyzed, they are mainly located in the intronic regions of cytokine genes. Although these SNPs do not directly alter the amino acid sequence, they are likely to affect cytokine expression through regulatory mechanisms. This is why genome-wide association analyses yielded results of modification associations between different traits and SNPs that can represent traits in terms of SNP specificity (25).

### Mendelian randomization

To investigate the causal relationship between inflammatory cytokines and IVDD, we performed an MR analysis. Causal effects were calculated by dividing the SNP-outcome effect by the SNP-exposure effect estimate. To contain multiple SNPs, various tests were performed, including inverse variance weighted (IVW), weighted median and MR-Egger. Cochrane’s Q tests were performed to assess heterogeneity between SNPs associated with each classification. In the presence of heterogeneity (*p* < 0.05), a random-effects IVW test was used to provide a more conservative but robust estimate. The weighted median test produced consistent estimates when ≥50% of the weights were from valid IVs. The MR-Egger regression test allowed for the presence of polymorphism in more than 50% of the IVs. To ensure the robustness of our MR analysis workflow, we have validated our methods against known examples from the literature. Specifically, we have referenced studies where MR has been successfully used to identify causal relationships between genetic variants and complex traits or diseases. For instance, the role of IL-18 in cardiovascular diseases and the involvement of IFN-γ in autoimmune disorders have been established through MR studies, demonstrating the efficacy of these methods in elucidating causal pathways (23). We have included a discussion on the error rates and the potential for false positives in our MR analysis. By employing robust statistical methods such as the Inverse Variance Weighted (IVW) method, MR-Egger regression, and the weighted median approach, we minimize bias and account for pleiotropy. Additionally, we performed sensitivity analyses and heterogeneity tests to ensure the reliability of our findings (26, 27).

All statistical analyses were performed using R (version 4.2.2). IVW, Weighted Median and MR-Egger regression methods were performed using the “TwoSampleMR” package (version 0.5.6). The MR-PRESSO test was performed using the “MRPRESSO” package. The MR-PRESSO tests were performed using the “MRPRESSO” (version 1.0) package.

## Results

### The effect of 41 inflammatory cytokines on IVDD

To obtain enough SNPs for the analysis, we set the threshold of P to *p*-value<5 × 10^−6^. After the MR analysis, we identified two inflammatory cytokines that are causally related to IVDD. The two inflammatory cytokines are: Interferon gamma (P_IVW_ = 0.000451007619315954 beta = −0.15507530020832 OR = 0.856350691670268) and IL-18 (P_IVW_ = 0.0169539375350741 beta = −0.0584055908393223 OR = 0.943267289).

**Figure 2:**
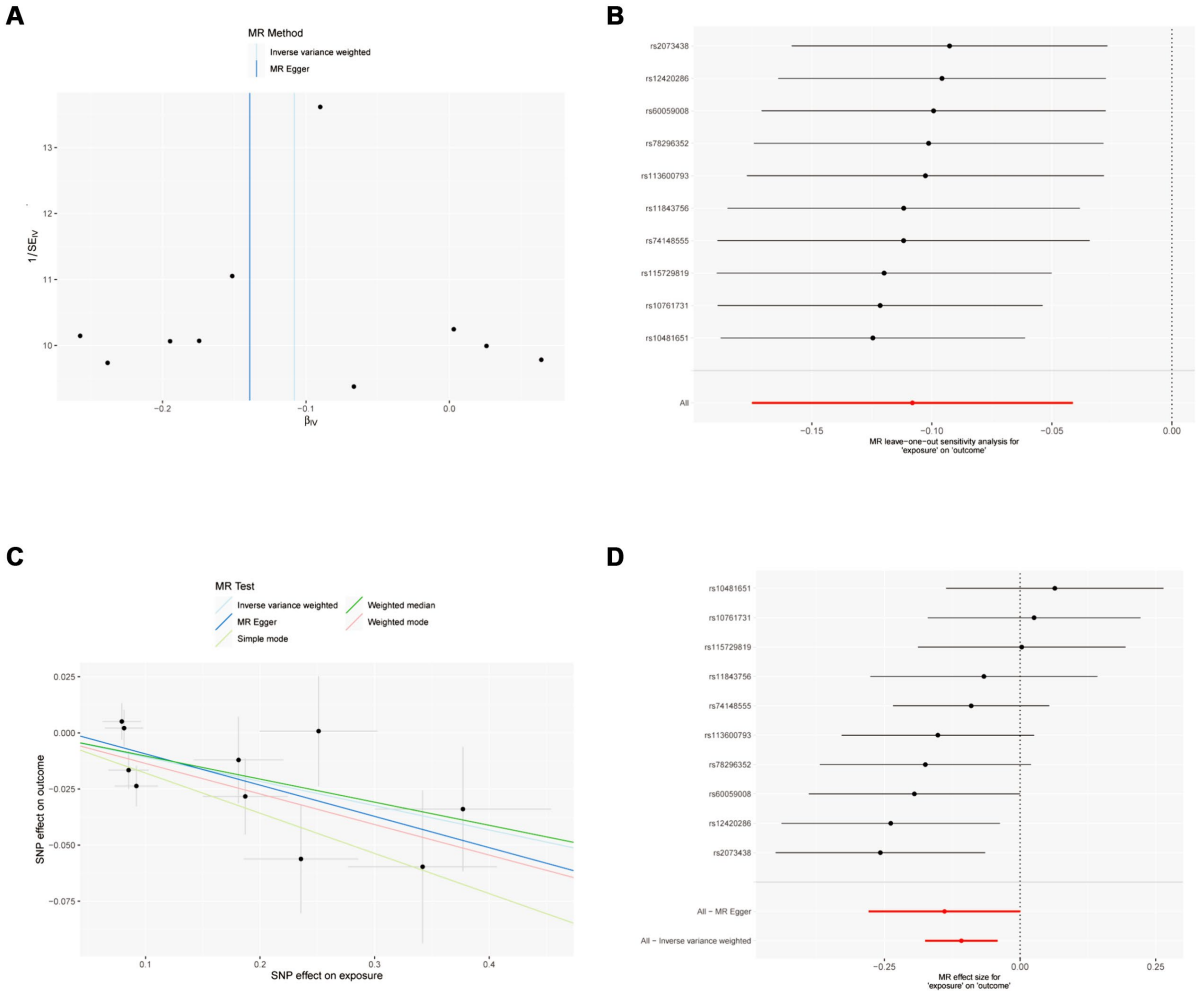
MR results of IFN-γ and IVDD: exposure = IFN-γ, outcome = IVDD. **(A)** Funnel plot of the causal effect of IFN-γ related SNPs on IVDD; **(B)** Plot of the leave-one-out analysis test of the causal effect of IFN-γ related SNPs on IVDD. **(C)** Scatter plot of genetic correlation between IFN-γ and IVDD by different MR methods. The slopes of the lines represent the causal effects of each method, respectively; **(D)** Forest plot of the causal effects of IFN-γ associated SNPs on IVDD.

**Figure 3:**
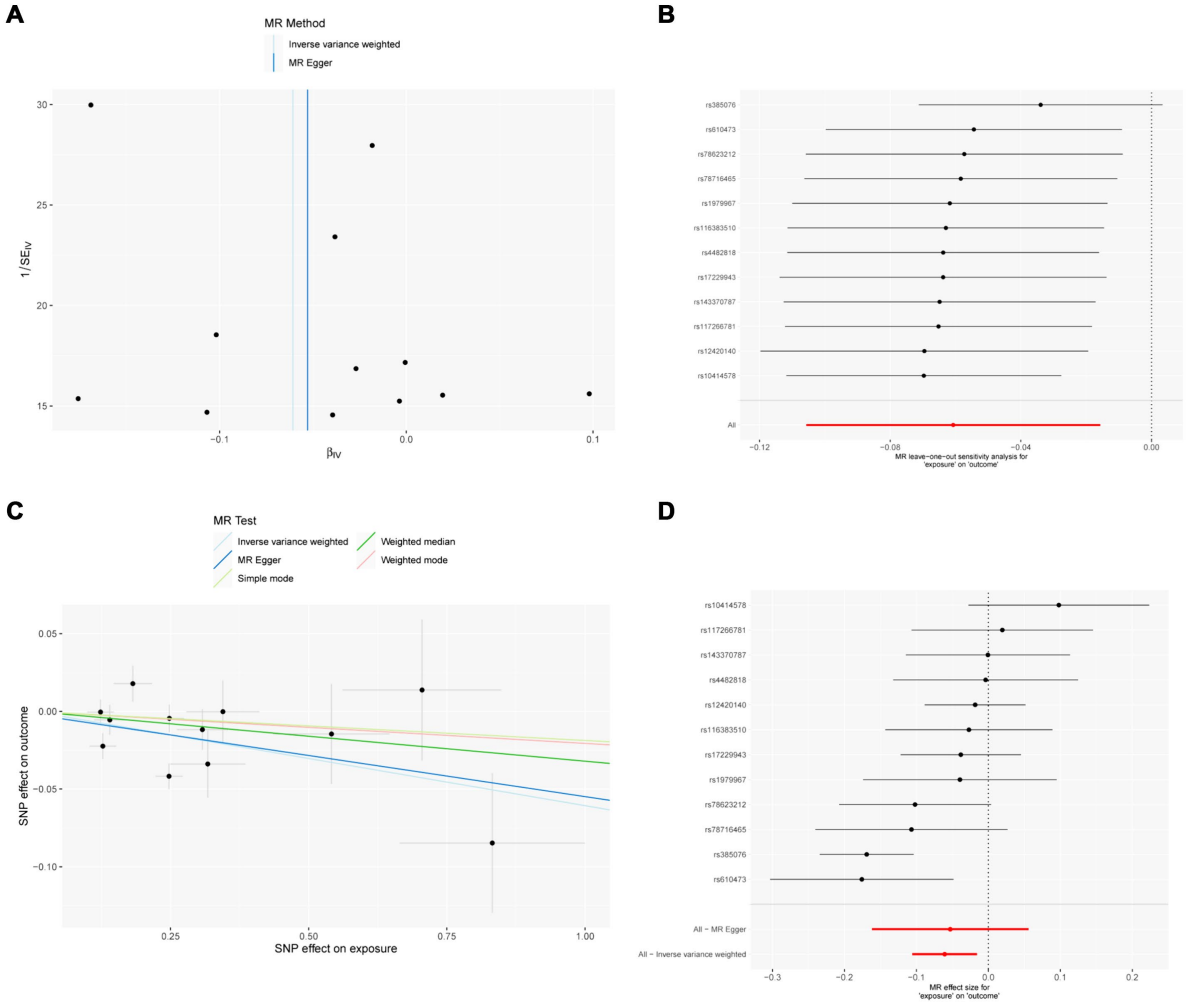
MR results of IL-18 and IVDD: exposure = IL-18, outcome = IVDD. **(A)** Funnel plot of the causal effect of IL-18 related SNPs on IVDD; **(B)** Plot of the leave-one-out analysis test of the causal effect of IL-18 related SNPs on IVDD. **(C)** Scatter plot of genetic correlation between IL-18 and IVDD by different MR methods. The slopes of the lines represent the causal effects of each method, respectively; **(D)** Forest plot of the causal effects of IL-18 associated SNPs on IVDD.

The sensitivity analysis of MR analysis included the test of pleiotropy, the test of heterogeneity, and the leave-one-out method. For the test of pleiotropy, we used MR-PROSS to detect gene-level pleiotropy, and confirmed the absence of horizontal pleiotropy when the *p*-value was greater than 0.05. In our study species, all the results were greater than 0.05, indicating no pleiotropy (Table 1). For the test of heterogeneity, we used Cochran’s Q test to detect heterogeneity, and confirmed the absence of heterogeneity when Q-value was greater than 0.05 (Table 2). The results of the study did not need to account for the effect of heterogeneity. The “leave-one-out” method involves removing each SNP step by step (Figures 2, 3), calculating the meta effect of the remaining SNPs, and observing whether the results change after removing each SNP. If the results change a lot after removing a SNP, it means that there is a SNP that has a great influence on the results, which is undesirable. Ideally, the results should not change much after removing each SNP step by step. In our analysis, the results did not change much at the overall error line after removing each SNP, which indicates the reliability of the results.

**Table 1:** Pleiotropy test in MR analysis results.

Outcome	Exposure	Egger intercept	SE	*p*-value
IVDD	IFN-γ	0.004552904	0.009046907	0.628345924
IVDD	IL-18	−0.001968729	0.012611993	0.879060651

**Table 2:** Detection of heterogeneity in MR analysis results.

Outcome	Exposure	Method	Q	Q_df	Q_pval
IVDD	IFN-γ	MR Egger	11.12655273	8	0.194631147
		Inverse variance weighted	11.47879982	9	0.244310826
	IL-18	MR Egger	26.24701561	10	0.00342179
		Inverse variance weighted	26.31097209	11	0.005832536

### Interferon gamma

MR analysis showed a negative causal relationship between interferon gamma and IVDD (Table 3; Figure 2). We also tested the results of the MR analysis for heterogeneity and Pleiotropy test (Tables 1, 2).

**Table 3:** Arithmetic results of IFN-γ and IVDD after MR analysis.

Outcome	Exposure	Method	NSNP	B	SE	*p*-value	lo_ci	up_ci	Or	or_lci95	or_uci95
IVDD	IFN-γ	Inverse variance weighted	10	−0.108	0.034	0.002	−0.175	−0.041	0.898	0.840	0.960
		MR Egger	10	−0.139	0.071	0.087	−0.279	0.001	0.870	0.757	1.001
		Weighted median	10	−0.103	0.043	0.017	−0.187	−0.018	0.902	0.829	0.982
		Weighted mode	10	−0.136	0.069	0.082	−0.272	0.000	0.873	0.762	1.000
		Simple mode	10	−0.179	0.077	0.046	−0.331	−0.027	0.836	0.718	0.973

### IL-18

MR analysis showed a negative causal relationship between IL-18 and IVDD (Table 4; Figure 3). We also tested the results of the MR analysis for heterogeneity and Pleiotropy test (Tables 1, 2).

**Table 4:** Arithmetic results of IL-18 and IVDD after MR analysis.

Outcome	Exposure	Method	NSNP	B	SE	*p*-value	lo_ci	up_ci	Or	or_lci95	or_uci95
IVDD	IL-18	Inverse variance weighted	12	−0.061	0.023	0.008	−0.106	−0.016	0.941	0.900	0.984
		MR Egger	12	−0.053	0.056	0.363	−0.162	0.056	0.948	0.851	1.057
		Weighted median	12	−0.032	0.024	0.187	−0.080	0.016	0.968	0.923	1.016
		Weighted mode	12	−0.021	0.033	0.542	−0.085	0.044	0.980	0.919	1.045
		Simple mode	12	−0.019	0.038	0.630	−0.093	0.055	0.981	0.911	1.057

## Discussion

In this study, we used MR analysis to investigate the causal relationship between 41 inflammatory cytokines and IVDD. We found that there was a significant causal relationship between IFN-γ as well as IL-18 and IVDD; that is, the chances of IVDD were greater in the presence of a decrease in the levels of IFN-γ and IL-18. Based on our study, we consider IFN-γ and IL-18 to be one of the upstream causes of IVDD pathogenesis. Specifically, SNPs associated with decreased cytokine levels were found to be significantly associated with increased risk of IVDD. This suggests a potential causal relationship between genetically determined reduced cytokine levels and increased susceptibility to IVDD. We need to emphasize the importance of mechanistic studies to determine the exact biological pathways behind these associations.

Previous investigators have extensively studied the role of inflammatory cytokines in the pathogenesis of IVDD. IL-1β, IL-6 Piezo1 and TNF-α are considered to be associated with IVDD (28–32). In addition, current anti-inflammatory therapeutic studies in IVDD focus on inhibition of inflammatory pathways (e.g., NF-κB) and downregulation of inflammatory factors and enzymes (e.g., IL-1β, TNF-α) (31). There are limitations to inferring causality from observational data (33, 34). These include untestable assumptions, chance causation and inverting causation (35). MR is a potentially robust method that can support this endeavor, and its scope for application will widen as the cost of data generation continues to reduce (23, 35). Findings from MR studies need to be interpreted in the context of other evidence related to the issue under investigation, and as such, it will contribute to the application of “inference to the best explanation” approaches to strengthening causal inference (36, 37).

IFN-γ, being the central effector of cell mediated immunity, can coordinate a plethora of anti-microbial functions (38). IFN-γ enhances antigen recognition through homologous T-cell interactions, increases the production of reactive oxygen species (ROS) and reactive nitrogen intermediates (RNIs), and induces an antiviral response (39, 40). This, in turn, expands antigen delivery through antigen-presenting cells (APCs) (41). Previous reports have suggested that A initiates oxidative stress mechanisms by inducing macrophage polarization, which may be a major mechanism in the pathogenesis of IVDD (42, 43). Our study, on the other hand, confirms the causal relationship between IFN and IVDD.

IL-18, a member of the IL-1 superfamily with a similar structure to IL-1β, is a highly regulated inflammatory cytokine that is cleaved by intracellular protease caspase-1 to generate a biologically active molecule (44). Previous studies have described the relationship between IL-1 as well as IL-1β and IVDD (44). IL-1 stimulates the production of several metalloproteinases, leading to connective tissue breakdown and inhibition of proteoglycans as well as type II collagen levels, thus having a global negative effect on articular cartilage (45). In addition, IL-1 exerts direct and indirect stimulatory effects on osteoclast maturation and is therefore involved in the development of arthritic bone erosion (46). Meanwhile, in previous studies, disc cells pretreated with IL-1β in response to FasL increased their apoptosis rate *in vitro* (47). Interestingly, our study found no causal relationship between IL-1 and IL-1β and IVDD. Instead, we suggest that IL-1 and IL-1β act through IL-18 in relation to IVDD. Specifically, IL-18 may affect endplate vascular endothelial cells, thereby altering the environment around NP cells, AF cells, and endplate chondrocytes (48).

Our attention is drawn to the fact that IL-18 was originally discovered as a factor that enhanced IFN-γ production from anti-CD3-stimulated Th1 cells, especially in the presence of IL-12 (49, 50). Therefore, we believe that IFN-γ and IL-18, as upstream factors in the pathogenesis of IVDD, do not exist independently of each other. However, there are no studies on their joint role in IVDD, which may provide a key to unraveling the biological mechanisms of IVDD.

A Genome-wide association study (GWAS) is a method used in genetics research to identify associations between specific genetic variants (such as single nucleotide polymorphisms or SNPs) and particular traits or diseases. It involves scanning the genomes of many individuals to look for genetic markers that occur more frequently in people with a certain trait or condition compared to those without it (51). GWAS can help researchers understand the genetic basis of complex diseases, uncover novel biological pathways, identify potential drug targets, and develop personalized treatment approaches (52).

There are several limitations to this study. 1. When we obtained the inflammatory cytokine data source, we noted that it still had a relatively small sample size compared to the GWAS data for other factors, even though it is already the largest inflammatory cytokine data currently available. Our findings may change if GWAS data from larger sample sizes for more countries, regions, ethnicities, etc. become available in the future. 2. In the screening step for IVs, we used *p* < 1.0 × 10^−6^ instead of the widely accepted *p* < 1.0 × 10^−8^ to obtain a larger number of SNPs, which in turn reduced the reliability of the results to some extent. This reduces the reliability of the results to some extent; after sensitivity analysis, our results are still plausible, but our conclusions need to be further validated by future studies.

## Conclusion

Our study identified a causal relationship between IFN-γ as well as IL-18 and IVDD. According to existing reports, potential mechanisms of action include inflammatory response and oxidative stress. As an emerging field of research, MR provides new perspectives to unravel the biological mechanisms of diseases and is valuable for disease monitoring and prevention as well as the exploration of targeted drugs. However, more evidence is needed to validate our study.

## Data availability statement

The original contributions presented in the study are included in the article/Supplementary material, further inquiries can be directed to the corresponding author. The analyses involved in this study were all completed in R via the TwoSampleMR-package.

## Ethics statement

Ethical review and approval was not required for the study on human participants in accordance with the local legislation and institutional requirements. Written informed consent from the patients/participants or patients/participants' legal guardian/next of kin was not required to participate in this study in accordance with the national legislation and the institutional requirements.

## Author contributions

FG: Writing – original draft, Writing – review & editing. CD: Data curation, Conceptualization, Formal analysis, Funding acquisition, Investigation, Methodology, Project administration, Resources, Software, Supervision, Validation, Visualization, Writing – original draft. ZW: Investigation, Conceptualization, Data curation, Formal analysis, Funding acquisition, Methodology, Project administration, Resources, Software, Supervision, Validation, Visualization, Writing – original draft. BW: Resources, Conceptualization, Data curation, Formal analysis, Funding acquisition, Investigation, Methodology, Project administration, Software, Supervision, Validation, Visualization, Writing – original draft. JL: Supervision, Conceptualization, Data curation, Formal analysis, Funding acquisition, Investigation, Methodology, Project administration, Resources, Software, Validation, Visualization, Writing – original draft. LS: Writing – review & editing, Conceptualization, Data curation, Formal analysis, Funding acquisition, Investigation, Methodology, Project administration, Resources, Software, Supervision, Validation, Visualization.

## Footnotes

^1^ https://data.bris.ac.uk/data/dataset/3g3i5smgghp0s2uvm1doflkx9x
